# Safety and efficacy of B-cell therapy in older people with multiple sclerosis

**DOI:** 10.3389/fimmu.2025.1699550

**Published:** 2026-01-28

**Authors:** Turlough Montague, Yuuki Kang, Karen Thomas, Nicole Burke Simpson, Celia Miller, Sophie Chatterton, Ariadna Fontes Villalba, John Parratt

**Affiliations:** Royal North Shore Hospital, Sydney, NSW, Australia

**Keywords:** anti-CD20 monoclonal antibodies, disease modifying therapies, immunosenescence, multiple sclerosis, ocrelizumab, ofatumumab, old age, PIRA

## Abstract

**Background:**

The MS population is aging, with nearly one-third now over 55 years. This group is underrepresented in trials and less often prescribed high-efficacy therapy (HET). Although phase III studies of ocrelizumab and ofatumumab confirmed efficacy in younger patients, the risk–benefit profile in older people with MS (opwMS) is less established given reduced relapse activity and higher risks of infection and malignancy.

**Methods:**

We retrospectively reviewed opwMS (≥ 55 years) treated with ocrelizumab or ofatumumab at a tertiary centre, evaluating clinical outcomes, MRI activity, and adverse events.

**Results:**

Among 140 patients (67% (94) female, mean age 63), median disease duration was 16 years and B-cell therapy duration 44 months; 91% (127) had prior DMT exposure. During treatment, 77.9% (109) achieved NEDA-3, EDSS remained stable (3.38 to 3.44, p=0.67), and PIRA was reduced by 15%. Adverse events occurred in 50.7% (71%), leading to discontinuation in 10.7% (15). Immunoglobulin levels fell significantly (IgM, IgA, and IgG). In 47 patients ≥ 65 years, adverse event rates were similar, 69.6% (33) achieved NEDA-3, but EDSS increased (p=0.034).

**Conclusion:**

Over 44 months, B-cell therapy in opwMS (mean age 63) showed safety and efficacy comparable with younger cohorts, although immunoglobulin decline and discontinuation due to adverse effects highlight the need for monitoring.

## Introduction

The average age of people with MS is increasing (1). Several factors contribute to this phenomenon including improved patient ascertainment, longevity, and an ageing population in high-prevalence regions (1, 2). Late-onset MS (LOMS), defined as symptom onset after 50 years, accounted for as many as 12% of incident cases in 2024 (3).

Despite the prevalence of opwMS, they are largely excluded from therapeutic trials (1). This is important as opwMS experience poorer relapse recovery and have a higher risk of progressive disease (4). A lack of disease-modifying therapy (DMT) efficacy and safety data might contribute to therapeutic inertia. A consensus statement published in Nature this year highlights the urgent need for improved understanding of the impact of ageing on all aspects of MS, including treatment in those over 50 years (5).

MS disease mechanisms shift with increasing age as demyelination from focal inflammatory activity decreases (6). Compartmentalised smouldering CNS inflammation persists within an intact blood–brain barrier giving rise to chronic active lesions (CALs) (7). These are encased in a rim of activated microglia and macrophages, typically occur after 10 years of disease, and peak at 50 years of age (7). Along with neuronal loss and brain and cord atrophy, they are considered a dominant driver of disability progression in opwMS (8).

The natural ageing of the immune system, referred to as immunosenescence, contributes to this changing disease profile, but its relationship to MS and DMTs is yet to be fully ascertained. Immunosenescence occurs because of cumulative antigenic stimulation and impaired pathogen clearance (9). Highly differentiated senescent T and B cells assume a different secretory profile that promotes a chronic low-grade inflammatory state, referred to as inflammaging (10). This occurs at a younger age in pwMS and can be seen within meningeal lymphoid follicles, with downstream chronic activation of microglia and macrophages that border CALs and contribute to neurodegeneration (11). Immunosenescence also disrupts CNS repair mechanisms, thereby impairing remyelination (11).

Ocrelizumab and ofatumumab are anti-CD20 monoclonal antibodies that deplete B cells. Ocrelizumab, a humanised mAb, is given via intravenous infusion every 6 months, whereas ofatumumab, a fully human mAb, is administered monthly subcutaneously. They bind distinct CD20 epitopes and differ in mechanism: Ocrelizumab predominantly mediates antibody-dependent cellular cytotoxicity, whereas ofatumumab mainly induces complement-dependent cytotoxicity (12).

Evidence supporting B-cell depletion in pwMS is largely based on the effect on relapse mechanisms and MRI lesions (13, 14). Although robust data are limited, the efficacy of B-lymphocyte suppression may be attenuated in opwMS. A 2017 meta-analysis of all DMTS predicted no efficacy after the age of 53 years in the average MS patient; however, this predates the era of B-cell therapy in MS (15). Considering studies in PPMS, wherein focal inflammatory lesions are less frequent, the treatment effect of ocrelizumab was greater in patients under 45 years in the ORATORIO trial (16). Similarly, rituximab showed no benefit in PPMS patients over 51 years without gadolinium-enhancing lesions (17).

However, clinical trials for ocrelizumab and ofatumumab in RRMS have demonstrated a benefit upon “progression independent of relapse activity” (PIRA) (18, 19). PIRA is responsible for at least 50% of all disability worsening in RRMS patients, and smouldering inflammatory mechanisms underpin this process (20). It is an important consideration when determining efficacy in older MS patients because PIRA events increase with age (20).

Five-year open-label extension data for ofatumumab showed fewer PIRA events in those who received ofatumumab continuously compared with patients that crossed over from teriflunomide (20). Ocrelizumab also proved superior to interferon beta 1a in preventing PIRA (19). The impact of B-cell depletion on CALs is not well established, and radiological studies report varied results (21, 22).

The risk of B-cell therapy in opwMS requires special consideration. Such patients are more vulnerable to serious infections and malignancy (5). They have higher levels of disability and comorbidity (5). More real-world evidence is urgently needed to better inform the risk–benefit assessment in this population.

## Methods

### Study population

Patients were identified from an MS clinic database at a tertiary referral centre in Sydney, Australia. Inclusion criteria were i) age ≥ 55 years, ii) treatment with ofatumumab or ocrelizumab for a minimum of 6 months, iii) a diagnosis of RRMS according to the 2017 revised McDonald criteria (23), iv) a minimum of 6-monthly clinic reviews by an MS specialist with formal EDSS assessment, and v) a minimum of one MRI brain and spine every 12 months.

### Study design

This is a single-centre, retrospective, cross-sectional study of all RRMS patients ≥ 55 years treated with ofatumumab and ocrelizumab to December 2023 at a tertiary MS referral centre. Ethics approval was obtained from the Northern Sydney Local Health District Human Research Ethics Committee.

A retrospective review of electronic medical records, MRI results, and pathology results from commencement of anti-CD20 mAb treatment was undertaken. Patient demographics, disease duration, Expanded Disability Status Scale (EDSS), and DMT history were recorded. EDSS was assessed 6-monthly. MRI scans of the head and spine were reviewed and collated with clinical data to determine three-parameter “No Evidence of Disease Activity” (NEDA-3) during treatment. Criteria for NEDA-3 were i) no relapse activity, ii),o EDSS increase (≥1 if EDSS < 5.5 and ≥0.5 if EDSS > 5.5), and iii) no new T2 or post-contrast T1 lesions on MRI. MRI scans of the brain and whole spine were routinely performed for a period of at least once per 12 month and reported by a neuroradiologist. They included T1, T2/FLAIR, and DWI sequences at a minimum. Post-contrast sequences were not required for inclusion, and contrast was given only when requested by the treating radiologist. Volumetric analysis was not performed.

Those who did not meet criteria for NEDA-3 were categorised as having relapse-associated worsening (RAW) or PIRA. RAW was defined as confirmed disability accumulation events characterised by new or worsening neurologic symptoms that were immediately preceded by a stable or improving neurological state for at least 30 days and were accompanied by objective neurological worsening and/or new T2/FLAIR or enhancing MRI lesions. PIRA was defined as an EDSS increase over 6 months of ≥1 if EDSS < 5.5 and ≥0.5 if EDSS > 5.5, without any evidence of relapse activity. People were determined to have secondary progressive disease if PIRA was sustained for greater than 12 months.

Patients underwent review of their lymphocyte subsets and immunoglobulin levels during B-cell therapy at non-fixed intervals. Hypogammaglobulinaemia was defined as IgG < 6 g/L. Lower limits of normal for IgM and IgA were 0.4 and 0.8 g/L, respectively. Those who did not have at least two blood results at an interval greater than 6 months were excluded from analysis of immunological parameters.

Adverse events were obtained from medical records. Mild injection or infusion related reactions were not included as these were inconsistently recorded. Adverse event and serious adverse event definitions were as per the Australian Therapeutic Goods Administration (24). Serious infections were defined as those requiring intravenous antibiotics, requiring hospital admission, or resulting in permanent organ dysfunction or death.

DMTs that were classified as high efficacy were ocrelizumab, ofatumumab, natalizumab, alemtuzumab, fingolimod, and cladribine.

### Statistical analysis

Statistical analysis was undertaken using SPSS version 30.0. The continuous outcomes are summarised with a mean and standard deviation (SD) when normally distributed and with a median and interquartile range (IQR) otherwise. Test selection was determined by variable type and distribution. Continuous variables were assessed for normality using histograms/Q–Q plots. Paired T tests were performed to assess change in immunological parameters. Logistic regression was performed to assess predictors of disease activity and infection. A multivariable model was used to obtain adjusted odds ratios (ORs) (95% CIs). The disease activity model adjusted for age, EDSS, and disease activity. The infection model adjusted for age, EDSS, and immunoglobulin levels where available. Assumptions were checked by design-based independence, Box–Tidwell tests for logit-linearity of continuous predictors (no significant interactions found), and VIF screening for multicollinearity from an equivalent linear regression model (all VIFs<1.1).

## Results

### Patient characteristics

**Figure 1:**
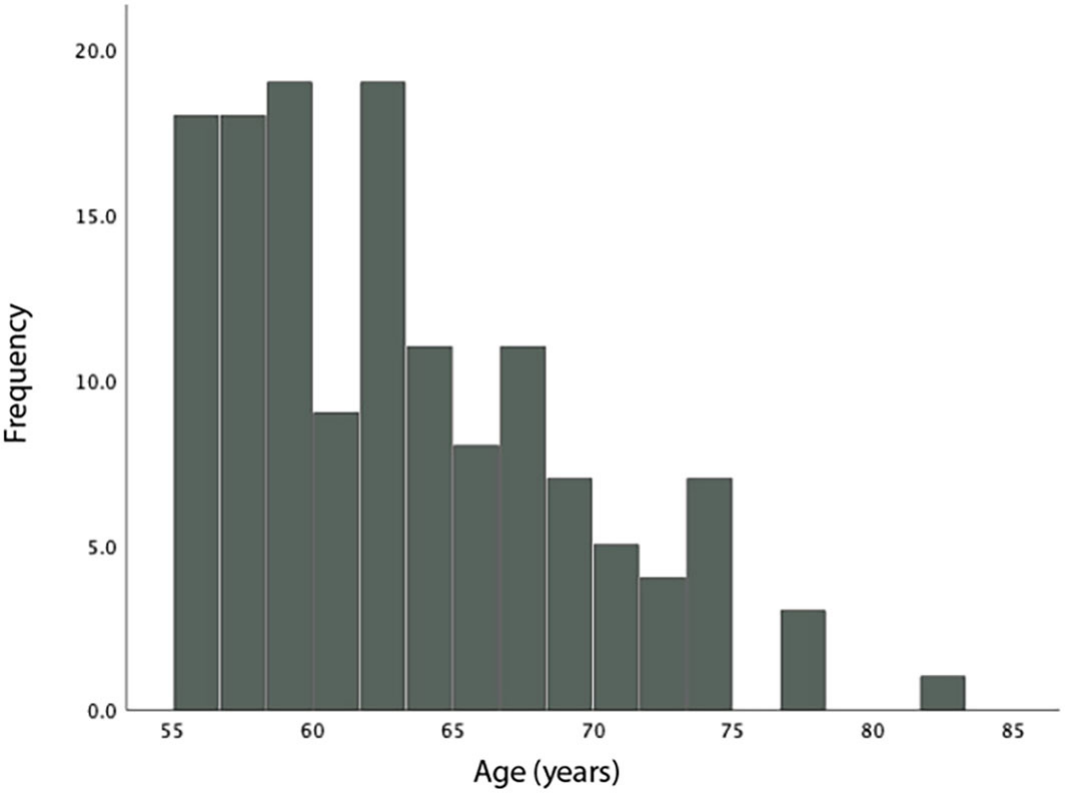
Age distribution.

**Figure 2:**
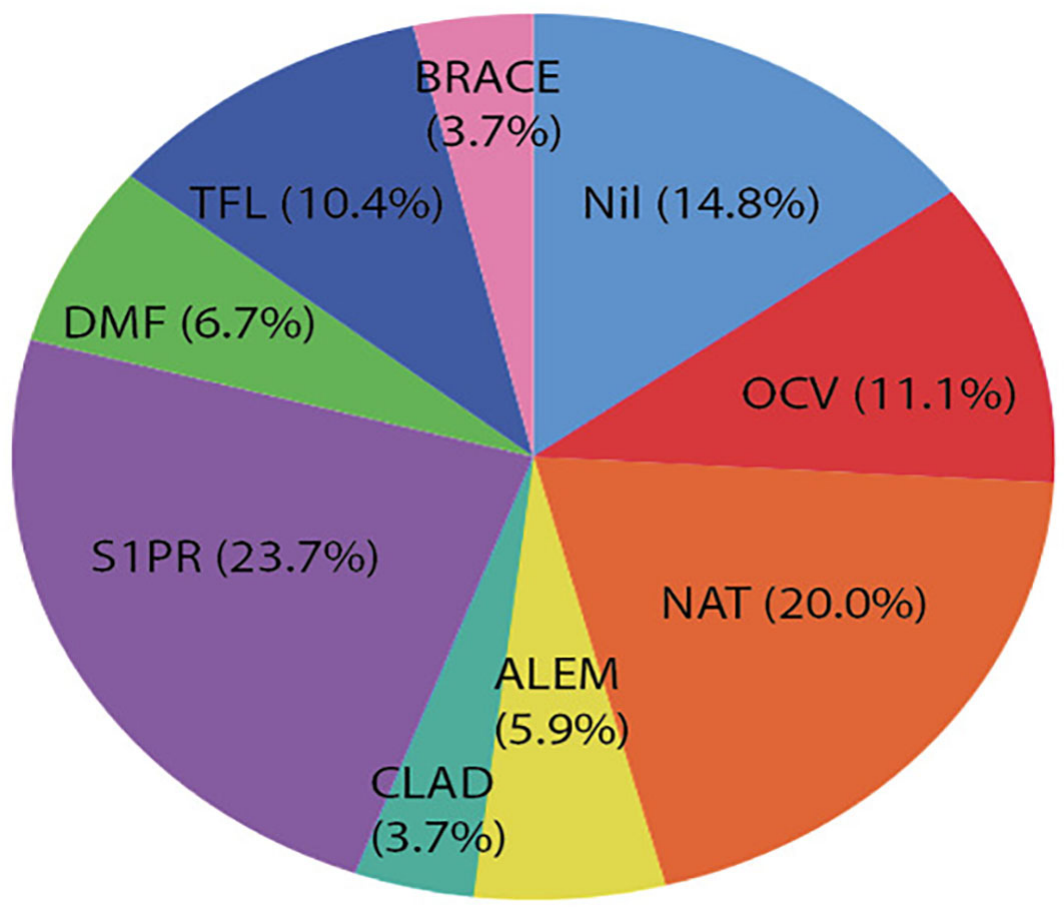
DMT prior to B cell therapy. Ocrelizumab (OCV), natalizumab (NAT), alemtuzumab (ALEM), cladribine (CLAD), shingosine-1-phosphate receptor modulator (S1PR), dimethyl fumarate (DMF), teriflunomide (TFL), and BRACE (Betaseron (interferon beta-1b), Rebif (interferon beta-1a), Avonex (interferon beta-1a), Copaxone (Glatiramer acetate), and Extavia (interferon beta-1b)).

A total of 140 patients were identified with a mean age of 63.1 (± 6.1) years (**Figure 1**). 67.4% (93) were women. The initial median EDSS was 3.0 (1.5-6.0) (**Table 1**). The median disease duration was 16 (11-26) years, and the median duration of B-cell therapy was 44 (26-77.5) months. 62.9% (88) were treated with ocrelizumab and 37.1% (52) with ofatumumab. 91% (127) were DMT-experienced prior to starting B-cell therapy, and 78.5% (110) switched from a high-efficacy DMT (HE-DMT) (**Figure 2**). In the 12 months prior to commencing B-cell therapy, 37.3% (52) demonstrated PIRA.

**Table 1:** Clinical characteristics of total cohort, ≥ 65 and <65 years.

Clinical characteristics	Total cohort (n=140)	≥ 65 years (n=47)	55-64 years (n=93)
Mean age (years)	63.1(± 6.1)	70.3 ( ± 4.1)	59.4 ( ± 2.8)
% Female	67.4	70.2	67.0
% ocrelizumab/% ofatumumab	62.1/37.9	59.6/40.4	63.5/36.5
Median disease duration (years)	16 (11-26)	19 (12-29.75)	14.5 (10.75-26.0)
Median initial EDSS	3.0 (1.5-6.0)	5.0 (2.13-6.4)	2.0 (1.13-4.0)
% DMT-experienced (% switched from HET)	91% (78.5%)	85.4% (70.7%)	94.6% (84.9%)
Median B-cell therapy duration (months)	44 (26-77.5)	39.5 (24-74.5)	54 (27.5-78.5)
Median number of ocrelizumab infusions/ofatumumab injections	12 (9-13)/23 (15-30)	11 (7–13)/22 (15-30)	12 (10-13)/24 (15 – 34)
% NEDA-3	77.7%	69.6%	82.6%
% disability improvement	12.9%	10.6%	15.2%

33.6% (47) of patients were ≥ 65 years. They had a mean age of 70.3 (*±* 4.1) years, and a mean EDSS of 4.1 (*±* 0.33) (**Table 1**). Their median treatment duration was 39.5 (24-74.5) months. 85.4% (40) of this age group were DMT experienced, and 70.7% (33) switched from an HE-DMT.

### Efficacy and safety

77.9% (109) of the total study population met the NEDA-3 criteria. A total of 29 opwMS had PIRA, and two had had relapse activity. In one, relapse was associated with enlargement of an existing and non-enhancing spinal cord lesion. Participants did not otherwise have new/enlarging T2 lesions or enhancing T1 lesions during the study. Seven people developed secondary progressive disease (mean age 62 (*±* 4) years). The proportion of patients with evidence of disease activity did not differ significantly between ocrelizumab (20.7%) and ofatumumab (24.5%) (p=0.6, 95% CI −0.18 to 0.10).

The mean age of patients with disability progression was 69.1 years. 12.9% (18) had disability improvement, and overall, the mean EDSS did not significantly increase during the study period (3.38 (*±* 2.27) to 3.44 (*±* 2.35), p=0.67).

In ≥ 65-year-old patients, 30.0% (14) had disability progression and 10.6% (5) had disability improvement. The mean EDSS significantly increased (4.1(*±* 0.33) to 4.3 (*±* 0.33), p=0.034).

Disease duration in years was a significant predictor of disease progression, whereas age was not. A longer disease duration by 1 year increased the odds of experiencing progression by 14.5% (p=0.03, CI 1.013 – 1.293).

**Figure 3:**
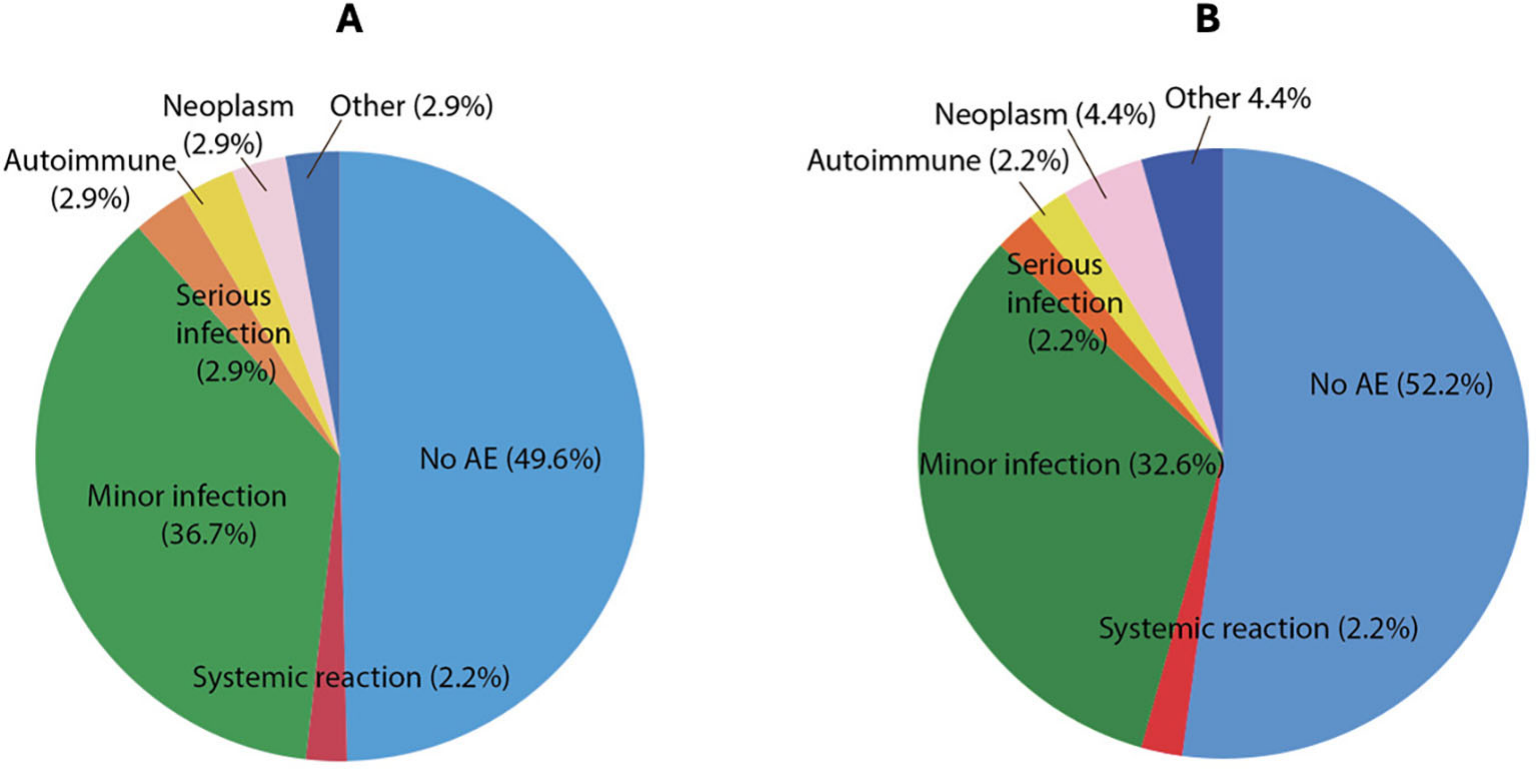
Adverse events AE in **(A)** total cohort **(B)** ≥65 years.

50.7% (71) recorded an AE (**Figure 3**), and in 15 (10.7%), B-cell treatment was stopped as a result. Indications for discontinuing treatment were infection (7), malignancy (3), secondary immune complications (3), and other (2). Serious adverse events occurred in 7 (5.7%) and included bacterial pneumonia, severe seronegative inflammatory arthritis, cryptogenic organising pneumonia, and malignancy. In the ≥ 65-year cohort, AE rates were similar, occurring in 46.8% (22) and 8.5% (4) stopping treatment as a result.

**Figure 4:**
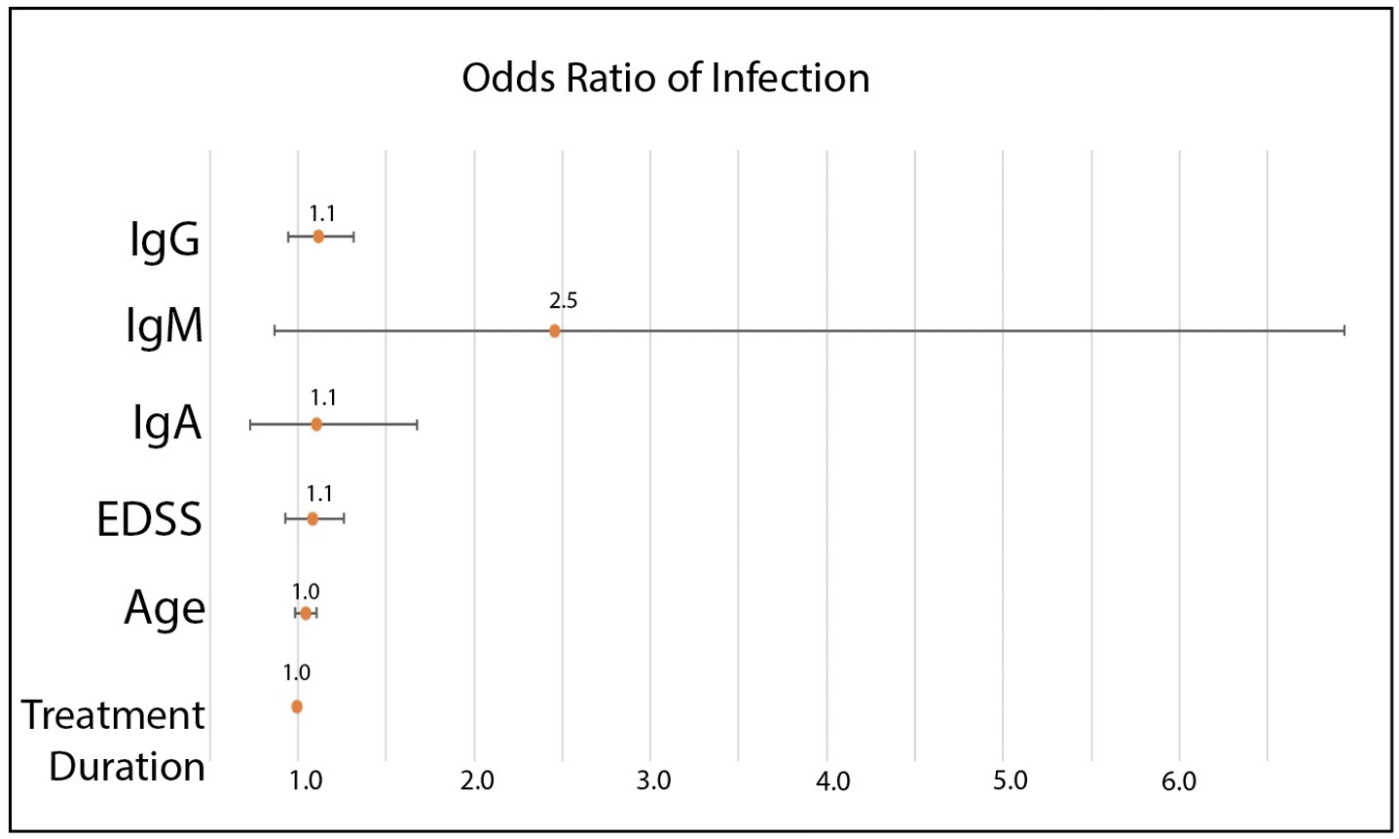
Odds ratio of infection with 95% confidence intervals. IgG OR 1.1 (95% CI 0.95-1.3), p=0.2. IgM 2.5 (95% CI 0.87-6.9), p=0.09. IgA OR 1.1 (95% CI 0.73-1.7), p=0.63. EDSS OR 1.1 (0.93-1.3), p=0.32. Age OR 1.0 (95% CI 0.98-1.11), p=0.99. Treatment duration OR 1.0 (95% CI 0.98-1.01), p=0.39.

Logistic regression was performed to determine the OR of infection (**Figure 4**) and disease progression. For infection, no parameter tested met significance, although a positive association was observed for lower immunoglobulins and higher EDSS. No association for age or treatment duration was found.

### Immunological parameters

A total of 106 patients with two blood results available on B-cell depleting therapy were included in analysis of immunoglobulins and lymphocyte subsets. The median times on treatment to first and second blood results were 13 (0.75-31) and 29 (9.5-64) months, respectively. The percentages of patients with immunoglobulins below the lower limit of normal were 32.1% (45), 9.3% (13), and 11.4% (16) for IgM, IgA, and IgG, respectively, at first sampling (**Table 2**). This increased to 39.3% (55), 10.8% (15), and 12.1% (17) at 29 months. The mean age of patients with hypogammaglobulinemia was 64.6 years. There was a significant decrease in all three parameters (IgM 0.68 to 0.57 (p<0.001), IgA 1.86 to 1.69 (p<0.001), IgG 8.8 to 8.5 (p<0.001)). Only 7 patients (6.6%) had a ≥ 20% decline in IgG, and their mean age was 60 years. The mean IgG values were significantly lower in the ≥ 65-year-olds compared with those aged 55–64 years (sample 1 p=0.021, sample 2 p=0.03). Measuring the effect of each drug on IgG individually, the percentage annual decrease for ofatumumab was 3.1% compared with 2.7% for ocrelizumab, which was not a significant difference (p=0.3).

**Table 2:** Trends in immunoglobulin levels.

Immunoglobulin	Mean Value (g/L)	% Below lower limit	% Below lower limit (≥ 65 years)	% Annual change	% Annual change (≥ 65 years)
IgG					
*Result 1*	8.8	11.2	18.9	-2.5 (± 5.9)	-2.2 (± 5.7)
*Result 2*	8.5	11.6	18.9		
IgM					
*Result 1*	0.68	32.1	37.8	-7.6 (± 12.2)	-5.2 (± 8.0)
*Result 2*	0.57	39.0	42.1		
IgA					
*Result 1*	1.86	9.4	10.8	-1.1 (± 11.2)	-0.83 (± 11.0)
*Result 2*	1.69	10.8	12.8		

CD8 and CD4 T cells and total lymphocyte count (TLC) significantly increased (CD8 0.31 to 0.36, p=0.02, CD4 0.71 to 0.82, p<0.001, and TLC 1.27 to 1.43, p<0.001).

### Comparison with phase III clinical trials

Compared with the pivotal trials for ocrelizumab and ofatumumab (13, 14), the mean age in this study is higher by approximately 25 years (63.0 ± 6.1 vs 37.2–37.9 years in OPERA/ASCLEPIOS) and patients had a higher baseline level of disability (EDSS 3.4 vs 2.8, **Table 3**). The median treatment duration was also longer (44 vs 21–24 months). Accounting for longer follow-up, adverse events causing treatment withdrawal and serious infection rates are comparable with ASCLEPIOS I and II, and slightly higher than OPERA I and II. The proportion of patients discontinuing treatment due to adverse events was 10.8%, corresponding to an OR of 3.36 (CI 1.72-6.30) compared with OPERA, and of 2.0 (1.09-3.62) compared with ASCLEPIOS. Compared with their respective open-label extension (OLE) studies, efficacy is slightly higher in this older cohort. 77% achieved NEDA-3 corresponding to OR of 1.78 (1.16–2.73) versus ocrelizumab OLE and OR 0.91 (0.60-1.37) versus ofatumumab OLE (25, 26).

**Table 3:** Comparison to phase III clinical trials and open label extension (OLE) studies.

Study	Median treatment duration (months)	EDSS	Age	AE causing treatment withdrawal	Serious infection	NEDA-3 (compared to OLE)
This study	44	3.4 (± 2.27)	63.0 (± 6.1)	10.8%	2.9%	77.7% (3.6 years)
ASCLEPIOS I&II	21	2.8 (± 1.3)	37.9 (± 9.2)	5.7%	2.54%	78.8% (4-year OLE)
OPERA I&II	24	2.8 (± 1.3)	37.2 (± 9.2)	3.2-3.8%	1.3%	65.4% (3-year OLE)

## Discussion

This retrospective analysis demonstrates the safety and tolerability of B-cell therapy in an older MS population with an average age of 63 years, 25 years older than pivotal clinical trials for ocrelizumab and ofatumumab. Over an extended follow-up period of 44 months, 10.7% (15) discontinued treatment due to adverse events and NEDA-3 rates are comparable with open-label extension studies in younger cohorts.

### Efficacy

In addition to advanced age, the study population had higher levels of disability compared with pivotal trials, and the majority switched from a different HET. PIRA was reduced by 15% in those experiencing it in the year prior to B-cell therapy. This suggests that B-cell therapy does have an effect on PIRA, even into older age. However, sub-analysis of patients ≥ 65 years demonstrates that the benefit attenuates and such patients had more PIRA events and EDSS progression. Logistic regression analysis did not demonstrate a significant association between increasing age and progression, which may relate to the small sample size of patients older than 65 years of age. Disease duration was found to be a significant predictor, with the odds of progression increasing by 14.5% for each additional year of disease, in keeping with the pathogenesis of compartmentalised and long-standing MS.

The study population has a lower-than-average EDSS for age compared with similar income countries. A recent German registry study found a mean EDSS of 4.2 in 55-64-year-olds and 5.3 in ≥ 65s, compared with 2.8 and 4.3 at the end of this study (1). HET use was only 38.5% and 25.4% for the two age groups in the German study (1). It is challenging to compare international cohorts, and we cannot exclude co-morbid factors, but these data may imply that HET contributes to lower disability even later in life.

This is supported by real-world studies demonstrating that HET significantly lowers PIRA risk, particularly when initiated early (27).

### Safety

Immunoglobulins significantly decreased during B-cell therapy, but lower levels were not significantly associated with increased infection.

Serum levels of IgG and IgM have been shown to significantly decline with healthy ageing, and age over 50 years is predictive of hypogammaglobulinemia in people on B-cell therapy (28, 29). Prevalence here was higher than in OPERA/ASCLEPIOS, acknowledging that those trials used a lower limit cut-off (5.65 versus 6.0 g/L in this study). Compared with a younger real-world ocrelizumab cohort (mean 48 years) over a comparable duration (3 years), the relative risk of hypogammaglobulinaemia in this study was 2.59 (1.22-5.48) (29), suggesting that older age is significantly associated with greater IgG decline.

Despite higher prevalence in ≥65s, IgG decline rates were similar across groups. Lower baseline IgG with ageing may explain this. Pretreatment levels were unavailable in this cohort. Importantly, infection rates were similar between age groups, and no associations with IgG, age, or treatment duration emerged, echoing data from a Canadian retrospective cohort study (30). In a real-world Italian cohort of 140 patients treated with ocrelizumab, IgG levels were not associated with infection over a median follow-up of 4.6 years. However, those over ≥55 years of age did have higher odds of infection (31).

Overall infection rates were lower than trials, partly due to recall bias in this retrospective design. Serious infection figures were robust given the single-centre tertiary setting. Adjusted for study duration, serious infection rates matched younger ASCLEPIOS/OPERA cohorts (12, 13). They are also aligned with long-term extension data from OLERO and ALITHIOS, in which serious infection rates were 2%-3% over 4–5 years of follow-up.

OpwMS are more vulnerable to serious infections regardless of DMT due to multi-morbidity, higher disability, and immunosenescence (5, 9).

This study population has a lower-than-average disability for age, which we propose protects against infection and partly accounts for the comparable serious infection rates to the younger trial populations. Notably, the ACAPELLA study of ocrelizumab did not find that infection rates increased when independently factoring in age over 55 years or EDSS over 6. However, patients meeting both these criteria did have a higher rate of infection (32). In the OPERA study, age, hypogammaglobulinaemia, and longer treatment duration with ocrelizumab were not associated with a higher risk of serious infections, whereas EDSS>6, co-morbidities, and recent relapse activity were (13). These findings, in combination with the low rate of serious infections in this study, suggest a good safety profile of B-cell therapy in opwMS that have reasonable mobility and minimal co-morbidity. Treatment decisions should be individualised, acknowledging that MS complications such as neurogenic bladder, bulbar dysfunction, and walking limitation will impact serious infection risk. This study reinforces the long-term positive effects of HET in reducing disability and consequently, mitigating against infections by reducing the neurological morbidity that contributes to them.

Infection rates here may also be impacted by the single-centre study design at a specialist MS centre. Accessible specialist nursing, comprehensive patient education, and early intervention for infections may have improved outcomes. Moreover, the local population’s life expectancy, 88.2 years for women and 85.4 years for men, is among the highest globally and may reflect broader health advantages (33).

## Conclusion

This retrospective analysis supports a favourable risk–benefit profile of ocrelizumab and ofatumumab in people with MS over 55 years, although the oldest patients (over 65 years) benefitted less. Serious infections were uncommon and comparable with rates in younger cohorts, which may have been influenced by lower disability and better general health given that IgG levels did reduce during the study. Overall, these findings will help to inform clinical decision making in ageing MS populations.

## Data Availability

The raw data supporting the conclusions of this article will be made available by the authors, without undue reservation.
